# Using Gagne’s instructional design to teach communication skills in phlebotomy education through role-play

**DOI:** 10.12688/f1000research.75335.2

**Published:** 2024-08-05

**Authors:** Amani Y. Owaidah

**Affiliations:** 1 Department of Clinical Laboratory Sciences, Imam Abdulrahman bin Faisal University, Dammam, College of Applied Medical Sciences,, Saudi Arabia

**Keywords:** communication skills, phlebotomy, medical education, trypanophobia

## Abstract

**Background:** Phlebotomy is a medical procedure that is performed frequently in the blood collection activities of medical institutions. The procedure involves close interaction with different types of patients—some of whom are cooperative and others, who, for many reasons, are not (for example, patients who have a fear of needles). Blood extraction is an essential skill in several medical specialties, such as in laboratory sciences. Lesson planning in phlebotomy education is mainly focused on procedural skills, and very little attention is given towards teaching communication skills despite the close patient interaction in phlebotomy. In this paper, I propose a lesson plan for teaching communication skills to medical laboratory sciences and nursing students based on Gagne’s instructional design.

**Methods:** The training session included two main parts: training session using Gange’s instructional design and at the end of the session, the participants were surveyed for the effectiveness of the training session.

**Results:** 17 participants were included in the study. Overall, the majority of the participants were highly satisfied with the effectiveness of the training session in teaching communication skills with all seven survey questions receiving a mean score of 4.58 on a Likert scale of 1-5.

**Conclusion:** We demonstrated the effectiveness of Gange’s instructional beyond theoretical lesson planning to teach communication skills through role-play in phlebotomy education.

## Introduction

Phlebotomy, also known as venipuncture, denotes the process of drawing blood, and it is considered one of the most common medical procedures in healthcare. ^1^ Often performed by doctors, nurses, laboratory staff, or specialized phlebotomists, it plays a pivotal role in all laboratory analyses, patient diagnoses, treatments, research, and transfusions. As many countries do not have specialized phlebotomy programs, the procedure is often embedded in the curriculum of most medical specialties. ^2^

In the field of medical education, phlebotomy has always been taught according to the apprenticeship model, which typically uses the “see one, do one, teach one” approach. ^3^ In particular, simulators such as mannequins, which offer a physical model of a patient’s arm, have been used to provide students the opportunity to practice a procedure before performing it on a real patient. ^4^ By using simulations the potential patient risk is reduced in clinical training, which ultimately improves the quality of patient care. Role-play is another teaching strategy that has been used. Students are generally divided into small groups and take turns practicing the steps related to the phlebotomy procedure. ^4^ These main teaching methods focus on the technical and procedural skills of phlebotomy, and neglect the vital skills of communication.

To perform the procedure in real-life (working) situations, healthcare professionals must interact with patients, some of whom may present anxiety or a fear of needles. ^5^^–^^7^ Other patients may be difficult to communicate with, especially when the first attempt to draw blood was unsuccessful. This may cause delays in extracting the sample or distress in the phlebotomy process. Therefore, teaching communication skills in phlebotomy is as vital as teaching technical skills.

Comprising nine steps, Gagne’s instructional design has been widely used in medical education as a framework for achieving five main learning outcomes (see Table 1), including intellectual skills, verbal information, cognitive strategies, attitudes, and motor skills. ^8^ In this study, the researcher applied Gagne’s steps to phlebotomy education and outlined a detailed lesson plan that incorporates the teaching of communication skills for the process of phlebotomy.

**Table 1.** Gagne’s nine steps of instructional design.

Step No.	Instructional step
1	Gain attention
2	Inform the learner of the lesson’s objectives
3	Recall prior knowledge
4	Present stimulus
5	Provide learning guidance
6	Elicit performance
7	Provide feedback
8	Assess performance
9	Enhance retention and transfer of acquired knowledge

*Note.* Adapted from Gagne’s *Principles of Instructional Design.* ^8^

## Methods

### Setting

In October 2021, a two-hour training session on communication skills based on Gange’s instructional design using role-play was conducted for the fourth year Clinical Laboratory Sciences (CLS) students and interns of CLS and Nursing programs in Imam Abdulrahman Bin Faisal University, Dammam, Saudi Arabia. An email invitation was sent to the students and interns via email. 17 volunteer participants responded to the invitation. The training session was directed by the author, who acted as the trainer for the entire session. A teaching assistant participated to play the role of the patient in the session.

### Ethical approval and consent for participation

The program and the study were approved by the ethics committee of Imam Abdulrahman bin Faisal University. All participants provided a signed written informed consent to participate in the training session and study, and the study protocol was approved by the institutional review board of Imam Abdulrahman Bin Faisal University (IRB-2021-03-359). The study was conducted in compliance with the Declaration of Helsinki.

### Eligibility criteria

Students who had essential prior knowledge of the pre-analytical variables that are associated with blood collection. These variables include patient variables (fasting), technique variables (patient identification), and specimen variables (anticoagulant specimen processing).

### Lesson delivery method: Gagne’s nine items of instructional design

***Gaining attention***

To engage students at the start of the session, the presenter (also called trainer) played a video demonstrating how a phlebotomist interacts with a child who comes for blood collection but is afraid of needles. This video will not only capture the attention of visual and auditory learners, but also triggers the learners’ personal experiences with blood collection. Audio-visual presentation also stimulates auditory learners through audio narration while, the visual learners found themselves engaged by the interactions in the video. Meanwhile, the kinesthetic learners were drawn towards the activities in the video. Meanwhile, through watching the video and based on the situated learning theory, the interaction between the child and the phlebotomist stimulates past memories and facilitates the students to reflect on their past experience and promotes professional growth. ^9^ In addition, the video potentially helped keeping the students focused on the objectives throughout the session through reducing the cognitive load.

***Informing the learner of the objectives***

To keep students engaged, the presenter asked the students what they thought about the video before stating the session’s objectives. This was intended to help students appreciate the learning objectives in real work situations. The objective of the session was for students to successfully acquire certain capabilities and skills, including how to approach and greet patients in a positive and friendly manner; communicate effectively with patients who exhibit fear and anxiety; and communicate effectively with cooperative patients.

***Recall of prior learning***

For students to benefit from the session, the trainer used the constructivism learning theory approach. It is based on allowing students to rely on their previous knowledge and personal experiences, which is then aligned with new information so that the students can construct their own visual interpretations of real work situations. ^10^ To achieve this purpose, the students were asked about their recent visits to an outpatient clinic or laboratory to perform blood collection. In particular, they will be presented with questions such as a) “ *Have you experienced fear from a blood collection procedure*”?; “ *Do you know someone, such as a family member or friend, with trypanophobia (phobia of medical procedures)?; and “If yes, how do phlebotomists deal with such a situation?”*; “ *What would you do differently if you were a phlebotomist?*” This way, the students were prompted to recall their previous learning experiences and were given approximately five minutes to reflect. This exercise engaged the students’ interpersonal intelligence.

***Presenting stimulus***

In this segment of the session, the trainer used role-play to deliver the intended content. Role-play is one of the most widely used teaching methods for the acquisition of communication skills in medical education. ^11^ Tables 2 and 3 outline the role-play scenarios that were presented in the session. The first scenario demonstrates how students should communicate with a cooperative patient, while the second scenario demonstrates how they should communicate with a patient displaying trypanophobia (fear of needles). To ensure that the students remain engaged in the session, the facilitator encouraged them again to recall their personal experiences of visiting a clinic for a blood test.

**Table 2.** Role-play scenario for a cooperative patient.

Scenario Element	Description
Venue	Phlebotomy area in a blood bank laboratory.
Role	1. Phlebotomist. 2. Adult patient.
Equipment	• Two chairs. • Phlebotomy table holding syringes, vacutainers, unlabeled blood collection tubes, and blood test request forms.
Background	The phlebotomy area in the blood bank is responsible for collecting blood samples from patients for laboratory testing in response to request forms from treating physicians. The patients come to the phlebotomy area with the request forms. The phlebotomist’s role is to identify the patients correctly and greet them in a friendly and polite manner.
Scenario	Patient: Enters the phlebotomy area with a request form for a blood test. Phlebotomist: Approaches the patient in a friendly manner, saying, “ *Good morning. My name is X. How are you today?*” Patient: Responds by saying, *“I am good, thanks.*” Phlebotomist: Asks, “ *What is your name?*” Phlebotomist then cross-checks the medical record card and request form, along with the medical record number on both forms. Phlebotomist: Smiles encouragingly while labeling the tubes, saying, *“Alright, I will take a few milliliters of blood for the blood test your physician requested.*” Patient: Agrees and lifts his or her sleeve for the procedure.
Objectives	• To demonstrate positive behavior. • To identify the patient correctly.
Procedure requirements for the student	• Greet the patient with a smile and friendly attitude. • Maintain eye contact. • Identify the patient correctly. • Explain the procedure to the patient. • Maintain a positive attitude throughout the procedure.

**Table 3.** Role-play scenario for a trypanophobic patient.

Scenario Element	Description
Venue	Phlebotomy area in a blood bank laboratory.
Role	1. Phlebotomist. 2. Adult patient.
Equipment	• Two chairs. • Phlebotomy table holding syringes, vacutainers, unlabeled blood collection tubes, and blood test request forms.
Background	The phlebotomy area in the blood bank is responsible for collecting blood samples from patients for laboratory testing in response to request forms from treating physicians. The patients come to the phlebotomy area with the request forms. The phlebotomist’s role is to identify the patients correctly and greet them in a friendly and polite manner.
Scenario	Patient: Enters the phlebotomy area with a request form for a blood test, displaying some hesitation. Phlebotomist: Approaches the patient in a friendly manner, saying, “ *Good morning. My name is X. How are you today*?” Phlebotomist: Notices the patient’s hesitation, asking, “ *Are you alright? Are you afraid of needles?*” Patient: Responds, saying, “ *Yes.*” Phlebotomist: Reassures the patient and maintains a comforting behavior, saying, “ *It is alright. I will use this tiny needle instead. How do you feel about that?*” The phlebotomist continues, asking, “ *Have you ever tasted a lemon before? Do you remember how uncomfortable it felt?*” Patient: Responds with a hesitant tone, saying, “ *Yes.*” Phlebotomist: Reassures the patient, saying, “ *It will feel just like that, and it will take less than a second.*” Patient: Responds, saying, “ *Alright.*” Phlebotomist: Continues procedure, saying, “ *My name is X. What’s yours?*” The phlebotomist then cross-checks the medical record card and request form, along with the medical record number on both forms. Phlebotomist: Smiles while labeling the tubes, saying, “ *Alright, I will take a few milliliters of blood for the blood test your physician requested, and I’ll use this tiny needle.*” Patient: Responds hesitantly, saying, “ *Alright.*” Phlebotomist: Instructs in a warm tone, saying, “ *Please pinch yourself in the arm for me. What did that feel like?*” Patient: Responds, saying, “ *It feels a bit uncomfortable.*” Phlebotomist: Responds, asking, “ *Did it hurt much?”* Patient: Responds, saying, “ *No.*” Phlebotomist: Responds, saying, “ *That is how this needle will feel.*” Patient: Responds with a sigh, saying, “ *Alright.*”
Objectives	• To demonstrate positive behavior. • To demonstrate compassion and reassurance. • To identify the patient correctly.
Procedure requirements for the student	• Greet the patient with smile and friendly attitude. • Identify the patient correctly. • Maintain comfortable eye contact. • Notice the patient’s fear. • Attempt to comfort the patient before explaining the procedure. • Be attentive to the patient’s needs. • Do not undermine the patient’s fear. • Explain the procedure to the patient. • Maintain a positive attitude throughout the procedure.

***Providing learning guidance***

For each role-play scenario, the trainer used Peyton’s approach to teaching, which comprises four steps: demonstration, deconstruction, comprehension, and performance. ^12^ For the first scenario (a normal, cooperative patient), the trainer began the instruction by demonstrating how students should approach the patient friendly and politely; identify the patient correctly; clearly explain the procedure; label the tubes accurately; and approach the patient for the phlebotomy procedure. Thereafter, the trainer deconstructed each step by explaining the rationale behind it. During deconstruction, students were encouraged to ask questions and comment on the process. By encouraging them to ask questions, the trainer facilitated their understanding, further stimulating them to reflect on previous experiences.

The second scenario (a trypanophobia patient) required a more explicit demonstration and deconstruction process. The same approach as in the first scenario was used, but the trainer demonstrated more compassion and persuasive skills when the patient (played by a teaching assistant) displayed signs of hesitation, fear, or discomfort. These signs of discomfort could be presented in different ways, such as patients with trypanophobia being hesitant to extend their arm for the procedure, or patients who start crying. The trainer, when taking the role of the phlebotomist, deliberately reacted to the patient’s anxiety by reassuring the patient and calmly asking, “ *Are you afraid of needles?*” Questions like these will show the patient that the phlebotomist is paying attention to his or her anxiety. Then, the trainer further reassured the patient by saying, “ *I understand that it might be scary, but I’m using this tiny needle, which looks just like a butterfly.*” Descriptive and calming statements like these will encourage the patient to recall associated childhood experiences (e.g., the quick and light brushes of butterfly wings). Subsequently, recalling these experiences can make the patients feel reassured that the short time of discomfort they feel is similar to what they remember, and that the procedure will be over quickly.

***Eliciting performance***

After the demonstration step was completed, the trainer conducted the performance step of Peyton’s approach. Students then were be divided into groups of three to role-play and practice their communication skills with different types of patients and were instructed to take turns performing as the phlebotomist, patient, and observer. This activity mimicked real work situations and helped students develop effective communication skills. The observers in the scenarios were responsible for witnessing the interactions between the phlebotomist and patient, as well as discussing the phlebotomist’s performance; they prompted discussions to explain what could be improved or done differently. Studies show that this step reveals the importance of role-play as a teaching method as it not only helps mimic real work situations, but also helps students work in teams and develop their communication skills. ^11^

***Providing feedback***

In addition to the observer’s feedback in the scenarios, the teaching assistant and trainer observed the students and provided immediate verbal feedback. Feedback, whether positive or negative, is an essential tool in education to help students achieve their maximum potential at any stage of their studies. ^13^ It should be constructive so that students are encouraged to improve their academic development. ^14^ The trainer used the tell, explain, listen, and let’ (TELL model) to provide feedback to students during the role-play performances, which comprises of four main features: tell (tell the students the specific behavior that requires improvement, or compliment them on their good behaviors); explain (offer explanations to show students the outcomes that might occur due to the behaviors demonstrated, whether positive or negative); listen (listen to and allow the students to reflect on the feedback that they have received); and ‘let’ (let the students know about the consequences of their actions if the behaviors are not corrected). ^14^

A common understanding among educators is that feedback is necessary only for the behaviors that require correction. However, feedback that focuses on areas of strength will help build students’ self-confidence and improve their communication with their superiors. ^15^ Overall, this step aimed to help students achieve the learning objective of the session—that is, to develop an effective communication skill set.

***Assessing performance***

**Figure 1.**
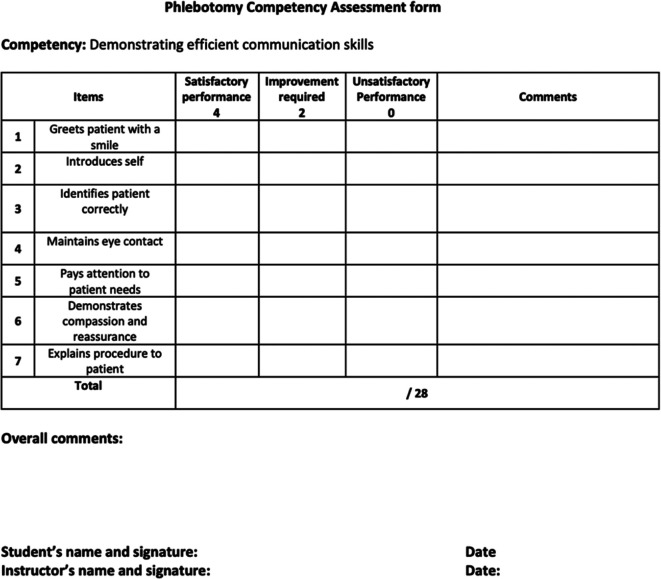
Assessment form.

After all groups took turns to perform each role and receive feedback, the students were regrouped into pairs. All students took turns to role-play as the phlebotomist and patient, while the teaching assistant and trainer observed how they interact in both roles. A formative assessment was used to evaluate the students’ performances and offered them the opportunity to reflect on and make the necessary adjustments to improve their future learning. ^16^ A graded checklist was used for the assessment and given to the students, so they would be better informed in future when performing the actions related to phlebotomy (see Figure 1).

***Enhancing retention and transfer***

The trainer used two methods to help students retain the knowledge that they gained and transfer it to others. First, 15 minutes before the end of the session, the trainer asked students to share their thoughts about the session, what they had learned, and to reflect on their performances. This approach allowed the students to not only retain their knowledge but also learn from the experiences of others. Second, the trainer provided the students their graded assessments to give them the chance to reflect on their performances privately.

Finally, at the end of the session, the facilitator provided a summary of the main learning objectives covered.

### Survey, data collection and analysis

At the end of the session, an online survey was sent to all participants to collect their views on the session. Answers to each question was given on a Likert scale of 1-5, where 5 meant strongly agree and 1 strongly disagree. The questions of the survey were modified from *Taniguchi et al* to fit the outcomes of the lesson plan. ^4^

The survey also included an open-ended question section to allow the participants to freely express their thoughts about the session. The open-ended section included three questions:

1. What did you learn from the session?
2. What did you like about the session?
3. What can you suggest to us to improve in the future?

The survey questions can be found in the *Extended data.*

The participants responses to the seven questions on the Likert scale 1-5 were analyzed using Microsoft excel to calculate the mean, median and interquartile ranges. The themes for the open-ended questions were identified in advance and the participants responses were categorized according to the pre-determined themes as follows: learning outcome, effectiveness of teaching strategy and areas for improvement.

## Results

15 out of 17 participants responded to the survey with a 88.2% response rate. All participants were females as the CLS and Nursing programs in Imam Abdulrahman bin Faisal University are offered only to females. Most of the participants were of the CLS specialty 93.75%, while only 6.25% were from the nursing specialty.

**Figure 2.**
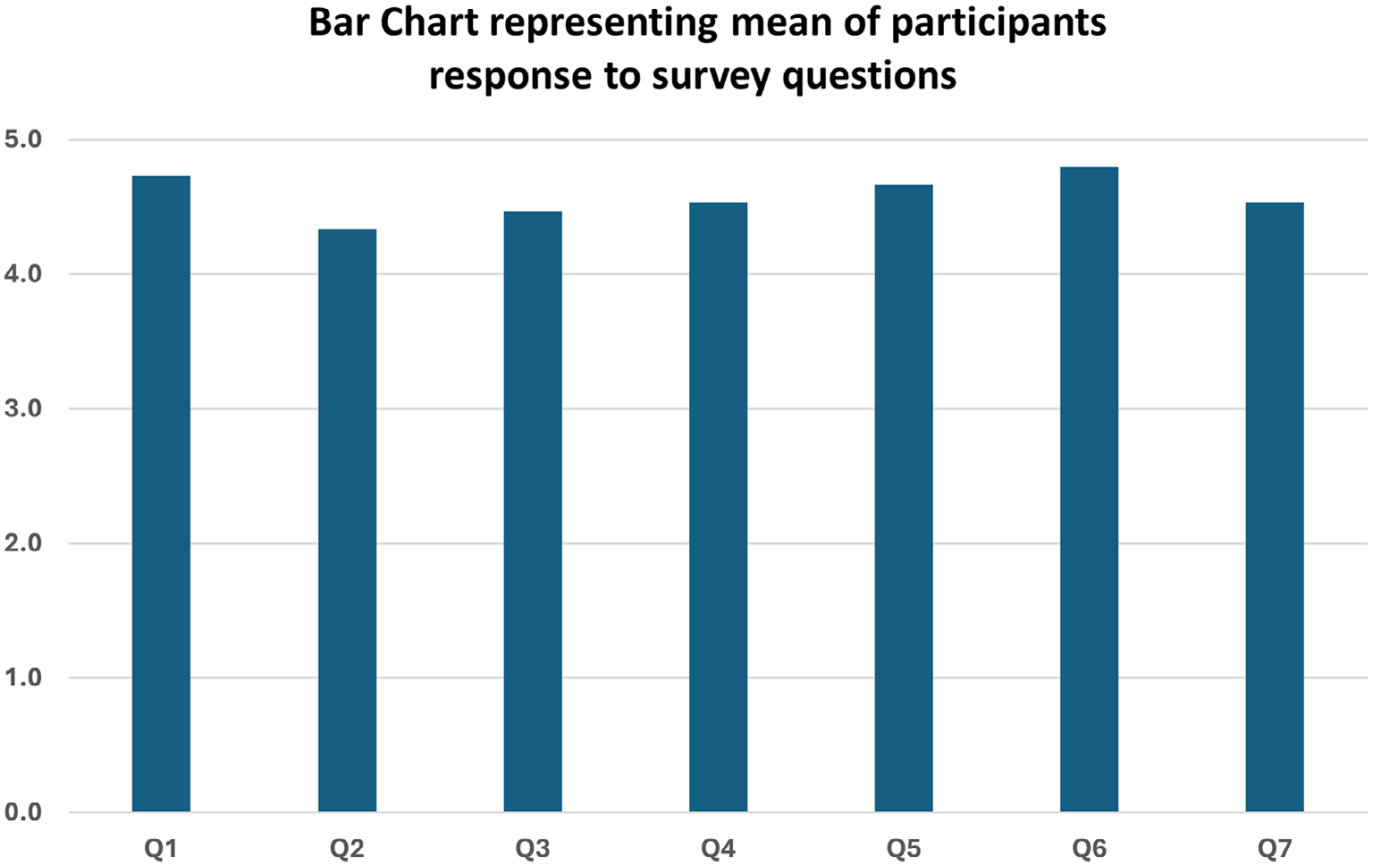
Bar chart representing mean of participants response to survey questions (n = 15). 5: strongly agree, 1: strongly disagree. **Q1:** Effectiveness of training session in teaching communication skills **Q2:** The video shown was suitable for demonstrating proper communication skills **Q3:** The case scenarios sufficiently addressed the communication problems with patients **Q4:** Provided an understanding of potential problems arising from blood collection **Q5:** Training session taught me patient management skills **Q6:** Training session helped me improve my communication skills **Q7:** Role-Play helped me improve my communication skills.

The survey questions are displayed in boxplots as seen in Figure 2. Generally, participant showed a high satisfaction to the training session. For example, a detailed question on the effectiveness of the session in teaching communication skills showed a mean score of 4.7 on the scale of 5, where 5 being the highest level of satisfaction.

10 out of 15 participants shared their thought about the session through answering the open-ended questions. The participants indicated that they learned how to listen and communicate with patients during phlebotomy, how to deal with different type of patients and most importantly to pay attention to detail. One participant said: “ *I learned possible situations I might be go into. Also how to pay attention to detail, and be transparent with the patient as much as possible.”* For the second question on what they liked about the session, the participants commended the creative teaching strategy using role-play as well as the interaction with their fellow participants and trainer demonstrating that they learned from listening to other participants sharing their experiences in phlebotomy “ *giving the opportunity to hear from everyone, listening to each story everyone had.*”

To improve the session in the future the participants suggested to add more videos and case scenarios for role-play and have a blind selection of role-play scenarios and dedicate more time for the session.

## Discussion

In this study, a lesson plan to teach communication skills in phlebotomy was developed and its effectiveness was assessed by the participants of the study. This session allowed the participants to practice their communication skills specifically during phlebotomy, where the focus of the curriculum in on the procedural skill despite that communicating with the patient is one of the crucial steps in ensuring a successful procedure. Overall, the participants were highly satisfied with the training session.

The result of this study supports other studies recognizing that incorporating role-play in educational programs is a valuable tool for medical students enabling them to practice multiple skill sets without patient risk. ^11^ Furthermore, the results shows that this type of teaching strategy has a great value to the trainees, it provided them with the opportunity to interact with other participants and learning from their previous experiences. This is important for professional growth as explained by the situated learning theory. ^9^ The participants responses to the open-ended questions addresses three main categories: learning outcome, effectiveness of teaching strategy and areas for improvement. 10 out of the 15 participants responding to the first question indicated that they have learned how to communicate with patients under different situations. This supports that the learning outcome of the session plan has been achieved. The responses on the participants on the effectiveness of teaching strategies supports the use of videos and role-play as an -educational teaching strategy to teach communication skills, which allowed them to be creative in visualizing the different scenarios they may face in the future. Under the category of areas of improvement, the participants suggested to include more role-play scenarios representing not only those with trypanophobia but different types of patients. In addition, to have a blind selection from a collection of scenarios not to limit it to two scenarios and to allow the participants to have a blind pick of multiple scenarios instead of taking turns to play a normal patient and a patient with trypanophobia. These responses could be utilized and invested improving educational curriculums. Various studies have demonstrated the benefits of co-creation of the curriculum, where students are seen as partners in education. ^17^^,^^18^

Despite the small number of participants in the study, it highlights the importance of communication skills in medical education as well as the importance of utilizing student’s responses in co-creation of the curriculum.

## Conclusion

In conclusion, in this study we have showed that Gagne’s instructional design does not only provide a platform for lecture-based lessons but can also be expanded to accommodate lesson planning for a wide range of skills, such as practical, communication, and interpersonal skills. The results also highlight the importance of students in the process of co-creation of the curriculum.

## Data availability

### Underlying data

Harvard Dataverse: Using Gagne’s Instructional Design to Teach Communication Skills in Phlebotomy Education through Role-Play. https://doi.org/10.7910/DVN/GTGMGE. ^19^

This project contains the following underlying data:

- Gange phelebotomy session data.tab

### Extended data

Harvard Dataverse: Using Gagne’s Instructional Design to Teach Communication Skills in Phlebotomy Education through Role-Play. https://doi.org/10.7910/DVN/GTGMGE. ^19^

This project contains the following extended data.

- Questionnaire phlebotomy education.rtf

Data are available under the terms of the Creative Commons Zero “No rights reserved” data waiver (CC0 1.0 Public domain dedication).

Due to the ethical and copyright limitations around social media data, the data from YouTube used in this study cannot be disclosed. Briefly, the video demonstrated the interaction between child and a phlebotomist. The child demonstrated her fear from needles by asking the phlebotomist multiple questions about the blood collection process and was pulling her arm away when the phlebotomist attempted to collect blood from her. The video showed how the phlebotomist communicated positively with the child explaining how the process is performed and trying to ease here worries and explaining to the child the consequences of moving her arm during blood extraction. The Methods section contains detailed information to allow replication of the study. Any queries about the methodology should be directed to the corresponding author.

## References

[1] Stedman TL : *Stedman’s medical dictionary for the health professions and nursing. A medical dictionary for the health professions and nursing.* 6th ed. Philadelphia, USA: Wolters Kluwer Health;2008.

[2] Mbah HA : Phlebotomy and quality in the African laboratory. *Afr J Lab Med.* 2014;3(1):4–7. 10.4102/ajlm.v3i1.132 29043181 PMC5637764

[3] Rodriguez-Paz JM Kennedy M Salas E : Beyond “see one, do one, teach one:” toward a different training paradigm. *Postgrad. Med. J.* 2009;85(1003):244–249. 10.1136/qshc.2007.023903 19520875

[4] Taniguchi JI Matsui K Araki T : Clinical training: a simulation program for phlebotomy. *BMC Med. Educ.* 2008;8:7. 10.1186/1472-6920-8-7 18221570 PMC2267176

[5] Cunha MLDR Brandi S Bonfim GFT : Application program to prepare child/family for venipuncture: experience report. *Rev. Bras. Enferm.* 2018;71(suppl 3):1474–1478. 10.1590/0034-7167-2017-0386 29972550

[6] Filbet M Larkin P Chabloz C : Barriers to venipuncture-induced pain prevention in cancer patients: a qualitative study. *BMC Palliat. Care.* 2017;16(1):5–7. 10.1186/s12904-016-0180-x 28095834 PMC5240299

[7] Woo WH : Using Gagne’s instructional model in phlebotomy education. *Adv. Med. Educ. Pract.* 2016; Volume7:511–516. 10.2147/AMEP.S110357 27621681 PMC5012615

[8] Gagné RM Briggs LJ Wager WW : Principles of instructional design. *Perform. Improv.* 2005;44:44–46. 10.1002/pfi.4140440211

[9] Knowles M : *The adult learner: a neglected species.* Houston, Texas: Gulf Publishing Company;1973 [cited 2021 April 19];211. Reference Source

[10] Dagar V Yadav A : Constructivism: a paradigm for teaching and learning. *Arts Soc Sci J.* 2016;7(4):66–70. 10.4172/2151-6200.1000200

[11] Nestel D Tierney T : Role-play for medical students learning about communication: guidelines for maximising benefits. *BMC Med. Educ.* 2007;7:3. 10.1186/1472-6920-7-3 17335561 PMC1828731

[12] Nikendei C Huber J Stiepak J : Modification of Peyton’s four-step approach for small group teaching—a descriptive study. *BMC Med. Educ.* 2014;14(1):68. 10.1186/1472-6920-14-68 24690457 PMC3976361

[13] Hesketh EA Laidlaw JM : Developing the teaching instinct, 1: feedback. *Med. Teach.* 2002;24(3):245–248. 10.1080/014215902201409911 12098410

[14] Hamid Y Mahmood S : Understanding constructive feedback: a commitment between teachers and students for academic and professional development. *J. Pak. Med. Assoc.* 2010;60(3):224–227. 20225784

[15] Brown N Cooke L : Giving effective feedback to psychiatric trainees. *Adv. Psychiatr. Treat.* 2009;15(2):123–128. 10.1192/apt.bp.106.003293

[16] Menéndez IYC Napa MAC Moreira MLM : The importance of formative assessment in the learning teaching process. *Int J Soc Sci Humanit.* 2019;3(2):238–249. 10.29332/ijssh.v3n2.322

[17] Lubicz-Nawrocka TM : Students as partners in learning and teaching: The benefits of co-creation of the curriculum. *International Journal for Students As Partners.* 2018;2(1):47–63. 10.15173/ijsap.v2i1.3207

[18] Bovill C Woolmer C : How conceptualisations of curriculum in higher education influence student-staff co-creation in and of the curriculum. *High. Educ.* 2019;78:407–422. 10.1007/s10734-018-0349-8

[19] Owaidah A : Using Gagne’s Instructional Design to Teach Communication Skills in Phlebotomy Education through Role-Play, Harvard Dataverse, V1, UNF:6:H0YiVGrIe1LxEYs1fFgEFA== [fileUNF]. 2021. 10.7910/DVN/GTGMGE PMC11462127 39386289

