# Supplementary material for: Using Gagne’s instructional design to teach communication skills in phlebotomy education through role-play
Source: F1000Res. 2024 Aug 5;11:46. Originally published 2022 Jan 14. [Version 2] doi: 10.12688/f1000research.75335.2 (PMC11462127; doi:10.12688/f1000research.75335.2)
Supplement: Peer review history

# Reviewer report: Reviewer response for version 2

**DOI:** 10.5256/f1000research.169898.r310989

**Published:** 2024-10-08

**Authors:** Conor Gilligan

**Affiliations:** 1 School of Medicine and Public Health, The University of Newcastle, Newcastle, NSW, Australia

I am happy with the responses and thank the authors for addressing my points.

Is the rationale for developing the new method (or application) clearly explained?

Partly

Is the description of the method technically sound?

Yes

Are the conclusions about the method and its performance adequately supported by the findings presented in the article?

Yes

If any results are presented, are all the source data underlying the results available to ensure full reproducibility?

Yes

Are sufficient details provided to allow replication of the method development and its use by others?

Yes

Reviewer Expertise:

NA

I confirm that I have read this submission and believe that I have an appropriate level of expertise to confirm that it is of an acceptable scientific standard.

# Reviewer report: Reviewer response for version 2

**DOI:** 10.5256/f1000research.169898.r321792

**Published:** 2024-09-10

**Authors:** Seetu Palo

**Affiliations:** 1 All India Institute of Medical Sciences, Hyderabad, India

The introduction provides a clear overview of the importance of phlebotomy and its teaching methodologies. The explanation of the apprenticeship model, simulation methods, and role-play is well-done. However, a brief explanation of why communication is so essential in phlebotomy, beyond just calming patient anxiety, would uplift the manuscript. While the article outlines Gagne’s nine steps and their application to phlebotomy education clearly, the justification for choosing Gagne’s framework is somewhat lacking. Providing more detail on why Gagne’s steps were chosen over other educational models would strengthen the article's theoretical foundation.

 In the methodology and results section, the use of videos and role-play is appropriate and well-explained, especially with respect to engaging different types of learners. The method of stimulating reflection through personal experiences adds depth to the learning experience. The methods section is comprehensive, but some details are missing. For instance, it would be beneficial to include a more precise explanation of the student’s pre-existing knowledge levels that might influence outcomes. The survey data presented is useful, but the analysis could be more robust. The article mainly reports the mean scores, but does not delve into any statistical tests that could indicate the significance of the findings. While the small sample size may limit this, even basic inferential statistics (e.g., confidence intervals) would provide more weight to the conclusions. The open-ended feedback from participants is valuable and provides qualitative insights, but, categorizing this data into themes is not explored in depth. Expanding on the key themes that emerged from the open-ended questions (e.g., learning outcomes, teaching effectiveness, areas for improvement) would enrich this section. The small sample size (n=17) is a major limitation of the study that should be acknowledged more explicitly.

 Overall, the article presents a valuable exploration of communication skills training in phlebotomy education, but it could be strengthened by further detailing the analysis of the findings and exploring the broader implications for medical education. Furthermore, the inclusion of practical suggestions for curriculum development and co-creation with students would enhance the impact of the study. The concept of students as partners in education is significant, but this article touches on it only briefly. If co-creation is an important take away from this study, it should be emphasized further with more explanation or examples from other medical education studies that support this approach. For instance, the suggestions made by participants (e.g., more videos, varied role-play scenarios) could be used as a basis for outlining future research directions or practical improvements to curriculum design.

Is the rationale for developing the new method (or application) clearly explained?

Partly

Is the description of the method technically sound?

Yes

Are the conclusions about the method and its performance adequately supported by the findings presented in the article?

Partly

If any results are presented, are all the source data underlying the results available to ensure full reproducibility?

Yes

Are sufficient details provided to allow replication of the method development and its use by others?

Yes

Reviewer Expertise:

Pathology, medical education

I confirm that I have read this submission and believe that I have an appropriate level of expertise to confirm that it is of an acceptable scientific standard, however I have significant reservations, as outlined above.

# Reviewer report: Reviewer response for version 1

**DOI:** 10.5256/f1000research.79193.r201321

**Published:** 2023-10-02

**Authors:** Ilona Heldal

**Affiliations:** 1 Western Norway University of Applied Sciences, Bergen, Norway

The paper explains the importance of explicitly utilizing a structured method (Gagne’s instructional design) for practicing communication and, within this, role-playing for learning phlebotomy. While the issue is important in many educational situations, unfortunately, I cannot suggest the paper for indexing. The aim is important, but it is difficult to understand the added value of the paper. 

 As the authors stated, it is vital to understand and learn how to practice communication skills in education, preparing students to deal with patients. Much of the obligatory moments are usually included in the procedural learning parts. Accordingly, I do not agree with “Lesson planning in phlebotomy education is mainly focused on procedural skills, and very little attention is given towards teaching communication skills despite the close patient interaction in phlebotomy.” Stated in the abstract. Additionally, the paper shows many similarities with the cited paper from Woo (Woo, W.H., 2016. Using Gagne’s instructional model in phlebotomy education. Advances in medical education and practice, pp.511-516.) without explaining fundamental differences from it.

 If the authors would like to index the paper, I suggest improving it by explicitly describing procedural learning, what within this learning can be achieved by Gagne’s method, and within this, again, how role-playing is important and planned. Here is a must to differentiate this study from Woo’s. Also, focusing on role-playing particularities, if the role-playing effect can be explicitly described and specificities of this case resulting in changes in student behavior would be a necessity.

Is the rationale for developing the new method (or application) clearly explained?

No

Is the description of the method technically sound?

No

Are the conclusions about the method and its performance adequately supported by the findings presented in the article?

No

If any results are presented, are all the source data underlying the results available to ensure full reproducibility?

Partly

Are sufficient details provided to allow replication of the method development and its use by others?

Yes

Reviewer Expertise:

Information systems. We have several projects on digital support of learning phlebotomy in various medical education.

I confirm that I have read this submission and believe that I have an appropriate level of expertise to state that I do not consider it to be of an acceptable scientific standard, for reasons outlined above.

## References

[1] : Using Gagne's instructional model in phlebotomy education. *Adv Med Educ Pract* .2016;7: 10.2147/AMEP.S110357 511-6 10.2147/AMEP.S110357 27621681 PMC5012615

# Reviewer report: Reviewer response for version 1

**DOI:** 10.5256/f1000research.79193.r201322

**Published:** 2023-09-12

**Authors:** Modibo Coulibaly

**Affiliations:** 1 Sominé DOLO hospital of Mopti, Mopti, Mali

Congratulations to the author of this paper. The manuscript is reasonably well written according to the scientific writing and meets the journal's requirements. In this paper, the author tried to emphasize the communication aspect in teaching phlebotomy through Gagne's pedagogical design and using role-playing games. This method adds a new skill to the routine of practitioners who had benefited from training focused on technical and procedural aspects.

 The implementation of this practice could contribute to improving the quality of patient care while focusing on their needs. The topic of this paper is in line with the scope of F1000research and would add value to scientific production.

 However, it contains some weaknesses that should be fixed.

 The term "blood extraction" is not too suitable. It should, therefore, be replaced by “blood draw” or “blood collection.”

 The author should give more detail on the study population by explaining why clinical laboratory sciences are offered only to women in his country.

 Regarding selecting participants, the author only announced that e-mails were sent to students and residents without specifying the number to which the e-mails were sent. So, it's difficult to determine the response rate and discuss other invitation methods, such as physically meeting to find participants.

 As worded in the paper, the response rate needs to be clarified. Did the ratio of 15 to 17 obtain it? In this case, the response rate is 88.2% instead of 94%. Thank you for specifying.

 The study concerns only trypanophobia, while there may be other phobias during phlebotomy, such as hematophobia ^1^, hypochondriacal patients ^2^, and nosocomephobia ^3^. To better understand the fundamental concern of patients when they appear anxious, the question to ask these patients must be an open question in the style of "What are you afraid of", instead of "Are you afraid of the needle"?

 Moreover, the role-play does not allow us to appreciate the real cognitive behavioral perceptions of the actual patients who could have particular intrinsic characteristics whose reflection on the solutions could improve the educational program.

 As indicated by the participants to include other types of patients, it would be relevant at this level to have patients of pediatric age. These evoke the double challenge and stress condition for the patient and the phlebotomist, given the difficulty of sampling in this category of patients.

 These conditions can only be assessed by associating a practical phase with real adult and child patients, inpatients, and outpatients.

 Finally, I suggest the author discuss integrating communication skills with the new approach to phlebotomy automation. Today, miniaturized sampling systems are based on combining ultrasound images, robotics, and artificial intelligence. Incorporating communication skills into these automated systems that perform safe and precise venous sampling would be a disruptive innovation in the sciences of phlebotomy ^4^.

Is the rationale for developing the new method (or application) clearly explained?

Yes

Is the description of the method technically sound?

Yes

Are the conclusions about the method and its performance adequately supported by the findings presented in the article?

Yes

If any results are presented, are all the source data underlying the results available to ensure full reproducibility?

Yes

Are sufficient details provided to allow replication of the method development and its use by others?

Yes

Reviewer Expertise:

Clinical Biochemistry

I confirm that I have read this submission and believe that I have an appropriate level of expertise to confirm that it is of an acceptable scientific standard, however I have significant reservations, as outlined above.

## References

[1] : Feasibility of self-organized blood sample collection in adults for study purposes in a primary care setting. *PLoS One* .2023;18(5) : 10.1371/journal.pone.0286014 e0286014 10.1371/journal.pone.0286014 37228048 PMC10212115

[2] : When Imagination Becomes a Disease: Dealing With Hypochondriacal Patients in Clinical Practice. *Ochsner Journal* .2019;19(2) : 10.31486/toj.18.0123 70-73 10.31486/toj.18.0123 PMC658419031258417

[3] : Prevalence of Nosocomephobia – Hospital Related Depression. *Annals of Psychophysiology* .2016;3(1) : 10.29052/2412-3188.v3.i1.2016.27-32 27-32 10.29052/2412-3188.v3.i1.2016.27-32

[4] : Design and Evaluation of a Handheld Robotic Device for Peripheral Catheterization. *J Med Device* .2022;16(2) : 10.1115/1.4053688 021015 10.1115/1.4053688 35284032 PMC8905093

# Response

**Published:** 2024-07-30

**Authors:** Amani Y. Owaidah

**Affiliations:** Imam Abdulrahman Bin Faisal University, Saudi Arabia

Dear Respected reviewer,

 Thank you very much for your valuable comments and feedback on our paper.

 Below is the response to each point you have raised. Looking forward to hearing from you in order to change version 2 accordingly.

**The term "blood extraction" is not too suitable. It should, therefore, be replaced by “blood draw” or “blood collection.”**

 The terms blood extraction are used interchangeably in laboratory sciences  with blood collection. However, the term is a matter of preference rather than an incorrect scientific term. It will be changed in version 2 to Blood collection based on your comments

**The author should give more detail on the study population by explaining why clinical laboratory sciences are offered only to women in his country.**

 In the university where the study was conducted the program is offered to only female as low enrollment rate were found in males so due to financial reasons the male section was closed.

**Regarding selecting participants, the author only announced that e-mails were sent to students and residents without specifying the number to which the e-mails were sent. So, it's difficult to determine the response rate and discuss other invitation methods, such as physically meeting to find participants. As worded in the paper, the response rate needs to be clarified. Did the ratio of 15 to 17 obtain it? In this case, the response rate is 88.2% instead of 94%. Thank you for specifying.**

 Thank you for pointing out the typo in the response rate. Indeed it is 88.2%. This will be changed accordingly. Yes, emails were the only method used to communicate with the participants. As mentioned in the paper that only level 4 students were invited to participate in the study therefore the emails of level 4 students was not difficult to track as the number of registered students are in the university’s data base.

**The study concerns only trypanophobia, while there may be other phobias during phlebotomy, such as hematophobia ^1^ , hypochondriacal patients ^2^ , and nosocomephobia ^3^ . To better understand the fundamental concern of patients when they appear anxious, the question to ask these patients must be an open question in the style of "What are you afraid of", instead of "Are you afraid of the needle"?**

 Thank you for suggesting other phobias. In the paper the author mentions trypanophobia as just an example which is the most commonly encountered. And the phrase “ **Are you afraid of the needle"?**

 Is used as a conversation opener rather than specifying a type of phobia. But this comment will be taken for future lessons.

**Moreover, the role-play does not allow us to appreciate the real cognitive behavioral perceptions of the actual patients who could have particular intrinsic characteristics whose reflection on the solutions could improve the educational program.**

 Role play does not assess the behaviour of actual patients. It is used only as a preliminary teaching methods for students practice. Simulation with real patients is definitely the next step in teaching to assess the real behavioral perceptions. But for the level of the students and administrative limitations simulations could not be used but could be a future study,

**As indicated by the participants to include other types of patients, it would be relevant at this level to have patients of pediatric age. These evoke the double challenge and stress condition for the patient and the phlebotomist, given the difficulty of sampling in this category of patients. These conditions can only be assessed by associating a practical phase with real adult and child patients, inpatients, and outpatients.**

 Agreed. As in the previous comment simulation with real patients could be a future study to conduct. Also, the reviewer could appreciate pediatric patients require a wide range of administrative process such as parental consent and IRB for involvement in a study.

 In addition the focus of the paper was for to use a different teaching strategy to just a procedural skill of blood collection. To help students improve their communication skills and teach them how to deal with different situations rather than just a procedure.

**Finally, I suggest the author discuss integrating communication skills with the new approach to phlebotomy automation. Today, miniaturized sampling systems are based on combining ultrasound images, robotics, and artificial intelligence. Incorporating communication skills into these automated systems that perform safe and precise venous sampling would be a disruptive innovation in the sciences of phlebotomy ^4^ .**

 This is an excellent point to consider for the future. However , these methods help in teaching procedural skills and the focus of our paper was communication skills. This point could be a focus of another paper using advanced methodologies to teach phlebotomy.

# Reviewer report: Reviewer response for version 1

**DOI:** 10.5256/f1000research.79193.r146424

**Published:** 2022-08-24

**Authors:** Conor Gilligan

**Affiliations:** 1 School of Medicine and Public Health, The University of Newcastle, Newcastle, NSW, Australia

The authors present a detailed description of their lesson plan for teaching communication skills associated with phlebotomy. The description of the lesson is clear and each step is well justified.

 The value of the focus on communication skills associated with phlebotomy is made clear, but the need for this work in terms of the application of Gagne's instructional design to this process is less clearly justified. There is a plethora of literature describing educational interventions for improving medical students' communication skills across diverse medical consultations. The authors could more clearly explain why the communication required during/associated with phlebotomy is unique and why a different teaching approach is required.

 The authors describe the lesson plan using language that presents the approach as very certainly achieve the desired outcomes. In many cases, these are in fact the intentions of the selected approaches, rather than absolutes.

 The authors refer learning styles in their 'gaining attention' section - presenting these as though they exist as entirely mutually exclusive ways of learning. Recent literature support the blurring of boundaries between learning styles. The approach used appropriately provides stimuli which will appeal to a range of learning styles and I think it would be useful to emphasize the blurred 'boundaries' between styles.

 I would like to better understand the logistics of the facilitator and teaching assisting observing and providing feedback on student role-plays - how was this managed with multiple groups and pairs conducting role-play at once?

 I'm not sure that a boxplot is the most appropriate format in which to present the data in figure 2 - perhaps a bar graph displaying means would be more clearly illustrative of the point?

 The limitations of this work need to be acknowledged. There is no measure of behavioural outcome. While it is recognised that such measures are difficult to achieve, it should be acknowledged that in the absence of such measures, the true effectiveness of this session remains unknown.

Is the rationale for developing the new method (or application) clearly explained?

Partly

Is the description of the method technically sound?

Yes

Are the conclusions about the method and its performance adequately supported by the findings presented in the article?

Yes

If any results are presented, are all the source data underlying the results available to ensure full reproducibility?

Yes

Are sufficient details provided to allow replication of the method development and its use by others?

Yes

Reviewer Expertise:

Medical education, interactional skills teaching, healthcare communication

I confirm that I have read this submission and believe that I have an appropriate level of expertise to confirm that it is of an acceptable scientific standard, however I have significant reservations, as outlined above.

# Response

**Published:** 2022-08-29

**Authors:** Amani Y. Owaidah

**Affiliations:** Imam Abdulrahman Bin Faisal University, Saudi Arabia

Dear respected reviewer,

 Thank you for your valuable comments that is highly appreciated. Following are my responses to your comments, to the best of my knowledge, I have addressed all the comments/ clarifications requested. 

**The value of the focus on communication skills associated with phlebotomy is made clear, but the need for this work in terms of the application of Gagne's instructional design to this process is less clearly justified. There is a plethora of literature describing educational interventions for improving medical students' communication skills across diverse medical consultations. The authors could more clearly explain why the communication required during/associated with phlebotomy is unique and why a different teaching approach is required.**

 With phlebotomy, the teaching approach is focused on the procedural skill of withdrawal of blood successfully, which is covered in m any laboratory sessions throughout the educational curriculum, however, with phlebotomy, there is a level of communication with patients/ donors with the phlebotomist. In real-life cases some patients present phobia of needles or anxiety, therefore, the phlebotomist with addition to excellent procedural skills has to have good communication skills to accommodate different types of patients in order to draw blood from them. In the absence of such skills the phlebotomist will not be able to ease patients and fulfill doctors request for testing.

**The authors describe the lesson plan using language that presents the approach as very certainly achieve the desired outcomes. In many cases, these are in fact the intentions of the selected approaches, rather than absolutes.**

**The authors refer learning styles in their 'gaining attention' section - presenting these as though they exist as entirely mutually exclusive ways of learning. Recent literature support the blurring of boundaries between learning styles. The approach used appropriately provides stimuli which will appeal to a range of learning styles and I think it would be useful to emphasize the blurred 'boundaries' between styles.**

 This is an excellent point to emphasize the different learning styles. However, the focus teaching strategy was not to go into detail on the various learning styles rather than presenting a well-known instructional  design for a lesson plan to follow to ensure proper implementation of  a new teaching approach. Follow up work could include the behavioral outcome on different cohorts.

**I would like to better understand the logistics of the facilitator and teaching assisting observing and providing feedback on student role-plays - how was this managed with multiple groups and pairs conducting role-play at once?**

  As the number of students were small. The instructor and teaching assistant moved around the groups while they were practicing their role-play to provide them feedback. In addition, as described in the paper the in the group one student took the role of the observer to observe the other two students. Which one played the patient and the other played the role of the phlebotomist. The observer also provided feedback to their colleagues on their performance. By this methods student gain the skill of peer assessment and raise their sense of self-awareness.  

**I'm not sure that a boxplot is the most appropriate format in which to present the data in figure 2 - perhaps a bar graph displaying means would be more clearly illustrative of the point?**

 The use of boxplots was selected by to present the different quartiles of the analysis. However, if necessary format could be presented in bar graphs.

**The limitations of this work need to be acknowledged. There is no measure of behavioural outcome. While it is recognised that such measures are difficult to achieve, it should be acknowledged that in the absence of such measures, the true effectiveness of this session remains unknown.**

 I agree, the behavioral outcome should be assessed to measure the true effectiveness of the session. However, However, the focus teaching strategy presenting a well-known instructional  design for a lesson plan to follow to ensure proper implementation of  a new teaching approach. Follow up work could include the behavioral outcome on different cohorts. Also could include feedback from clinical instructors to provide feedback on the effectiveness of this teaching method. The outcome of the current paper could be used to improve the design of the current curriculum by providing the students with the opportunity to co-create the curriculum.

 Looking forward to hearing from you
